# Insulin receptor substrate-1 prevents autophagy-dependent cell death caused by oxidative stress in mouse NIH/3T3 cells

**DOI:** 10.1186/1423-0127-19-64

**Published:** 2012-07-12

**Authors:** Shih-Hung Chan, Ushio Kikkawa, Hidenori Matsuzaki, Jyh-Hong Chen, Wen-Chang Chang

**Affiliations:** 1 Institute of Clinical Medicine, College of Medicine, National Cheng Kung University, Tainan, Taiwan; 2 Department of Internal Medicine, National Cheng Kung University Hospital, College of Medicine, National Cheng Kung University, 138 Sheng-Li Road, 704, Tainan, Taiwan; 3 Biosignal Research Center, Organization of Advanced Science and Technology, Kobe University, 1-1 Rokkodai-cho, Nada-ku, Kobe, 657-8501, Japan; 4 Department of Hygiene, Kawasaki Medical School, 577 Matsushima, Kurashiki, 701-0192, Japan; 5 Department of Pharmacology, College of Medicine, National Cheng Kung University, Tainan City, Taiwan; 6 Graduate Institute of Medical Sciences, College of Medicine, Taipei Medical University, Taipei City, Taiwan

**Keywords:** Insulin receptor substrate, Oxidative stress, Autophagy, Cell death, Cancer, Mammalian target of rapamycin, p70 ribosomal protein S6 kinase, Reactive oxygen species, Glucose oxidase

## Abstract

**Background:**

Insulin receptor substrate (IRS)-1 is associated with tumorigenesis; its levels are elevated in several human cancers. IRS-1 protein binds to several oncogene proteins. Oxidative stress and reactive oxygen species (ROS) are involved in the initiation and progression of cancers. Cancer cells produce greater levels of ROS than normal cells do because of increased metabolic stresses. However, excessive production of ROS kills cancer cells. Autophagy usually serves as a survival mechanism in response to stress conditions, but excessive induction of autophagy results in cell death. In addition to inducing necrosis and apoptosis, ROS induces autophagic cell death. ROS inactivates IRS-1 mediated signaling and reduces intracellular IRS-1 concentrations. Thus, there is a complex relationship between IRS-1, ROS, autophagy, and cancer. It is not fully understood how cancer cells grow rapidly and survive in the presence of high ROS levels.

**Methods and results:**

In this study, we established mouse NIH/3T3 cells that overexpressed IRS-1, so mimicking cancers with increased IRS-1 expression levels; we found that the IRS-1 overexpressing cells grow more rapidly than control cells do. Treatment of cells with glucose oxidase (GO) provided a continuous source of ROS; low dosages of GO promoted cell growth, while high doses induced cell death. Evidence for GO induced autophagy includes increased levels of isoform B-II microtubule-associated protein 1 light chain 3 (LC3), aggregation of green fluorescence protein-tagged LC3, and increased numbers of autophagic vacuoles in cells. Overexpression of IRS-1 resulted in inhibition of basal autophagy, and reduced oxidative stress-induced autophagy and cell death. ROS decreased the mammalian target of rapamycin (mTOR)/p70 ribosomal protein S6 kinase signaling, while overexpression of IRS-1 attenuated this inhibition. Knockdown of autophagy-related gene 5 inhibited basal autophagy and diminished oxidative stress-induced autophagy and cell death.

**Conclusion:**

Our results suggest that overexpression of IRS-1 promotes cells growth, inhibits basal autophagy, reduces oxidative stress-induced autophagy, and diminishes oxidative stress-mediated autophagy-dependent cell death. ROS-mediated autophagy may occur via inhibition of IRS-1/phosphatidylinositol 3-kinase/mTOR signaling. Our data afford a plausible explanation for IRS-1 involvement in tumor initiation and progression.

## Background

The insulin receptor substrate (IRS) proteins are a family of cytoplasmic adaptor proteins recognized for their role in insulin signaling. IRS-1 was the first of these to be identified as a 185 kDa protein that is detectable by immunoblot analysis in response to insulin stimulation [1]. IRS-1 shows no intrinsic enzymatic activity and contributes to signaling through its role as an adaptor for the organization of signaling complexes [2]. Upon activation by its upstream stimulators, IRS-1 generates binding sites for downstream effectors in its C-terminal region [3]. The main IRS-1 downstream signaling pathways include type I phosphatidylinositol 3-kinase (PI3K)/Akt (PKB: protein kinase B), mammalian target of rapamycin (mTOR), and mitogen activated protein kinase (MAPK)/extracellular signal-regulated kinase (ERK). Many of these effector pathways have been implicated in cell growth, proliferation, tumorigenesis, and cancer progression [4]. IRS-1 exhibits increased expression in hepatocellular, pancreatic, prostatic, breast, and ovarian cancers [5-10]. The activation of both MAPK and PI3K signaling pathways has been implicated in the stimulation of proliferation by IRS-1 [11].

Organisms living in an aerobic environment require oxygen for their vital cellular processes. Cells generate partially reduced forms of oxygen, collectively referred to as “reactive oxygen species” (ROS), during respiration and enzymatic processes. The production of ROS in excess of the organisms endogenous cellular capacity for detoxification and utilization results in a non-homeostatic state referred to as “oxidative stress” [12]. Low levels of ROS can promote cell proliferation but high levels induce cell death [13]. ROS and oxidative stress have long been associated with cancer. Cancer cells produce higher levels of ROS than normal cells do, due to increased metabolic stresses [14]. Additionally, ROS is involved in the initiation and progression of cancers, damage to DNA, genetic instability, cellular injury, and cell death [15-17]. Hence, the association of ROS with cancer cells is complex; it is important to understand how cancer cells can grow rapidly and survive while exposed to high levels of ROS.

Modes of cell death are usually defined by morphological criteria, and these include apoptosis, necrosis, autophagic cell death, mitotic catastrophe, anoikis, excitotoxicity, Wallerian degeneration, and cornification [18]. Oxidative stress induces apoptosis, and the molecular mechanisms involved have been well delineated [19]. Oxidative stress also induces necrotic cell death [20-22], and ROS was recently reported to induce autophagy [23-26] and apoptosis-independent autophagic cell death [27]. One molecular mechanism for oxidative stress-induced autophagy involves the activation of AMP-activated protein kinase (AMPK) [28]. AMPK is an upstream regulator of mTOR, the core negative regulator of autophagy [29], and it negatively regulates mTOR either by direct inhibition [30,31] or by activating tuberous sclerosis complex proteins, upstream negative regulators of mTOR [32]. Oxidative stress activates AMPK by stimulation of ataxia-telangiectasia mutated protein (ATM), an upstream activator of AMPK [33]. Taken together, oxidative stress can induce autophagy via AMPK-mediated inhibition of mTOR. Further, oxidative stress inhibits IRS-1/PI3K/Akt signaling via AMPK-dependent phosphorylation of IRS-1 at Ser-794, leading to dissociation of IRS-1 from its upstream membrane growth factor receptors [34]. Oxidative stress also reduces endogenous IRS-1 levels [34,35]. Because IRS-1/PI3K/Akt signaling can activate mTOR activity [31,36], which is well known to inhibit autophagy [31,36], it is possible that oxidative stress induces autophagy via AMPK-mediated inhibition of IRS-1/PI3K/Akt/mTOR signaling. By contrast, Akt inhibits AMPK by interrupting with its activation by liver kinase B (LKB)-1 [37]. Hence, it is possible that IRS-1 negatively regulates autophagy through Akt, to inhibit AMPK or to increase mTOR activity. However, although this appears to be a reasonable hypothesis, there have been no reports supporting the notion that increased levels of IRS-1 inhibit autophagy, until now.

Inevitably, ROS concentrations increase during rapid cell growth, and the increased ROS levels may kill the cells. ROS induces autophagy, which contributes to oxidative stress-mediated autophagic cell death [27], while both ROS and IRS-1 signaling can influence each other. Thus, we propose that IRS-1 plays an important role in oxidative stress-mediated autophagic cell death. In this study, we demonstrate that overexpression of IRS-1 promotes cells growth, inhibits basal autophagy, reduces oxidative stress-induced autophagy, and diminishes oxidative stress-mediated autophagy-dependent cell death. In addition, we provide evidence to support the notion that oxidative stress-induced autophagy may occur via inhibition of IRS-1/PI3K/mTOR signaling.

## Methods

### Cell lines

Cells overexpressing IRS-1:

Human IRS-1 (NM_005544) cDNA was cloned from a cDNA library and subcloned into pMXs retroviral vector (Cell Biolabs). The retroviral packaging cell line, Platinum-E cell line (Cell Biolabs), was then transfected with control pMXs vector or that containing human IRS-1 cDNA, using FuGENE 6 transfection reagent (Roche Applied Science). Retroviruses were harvested and used to infect NIH/3T3 cells using polybrene (Sigma-Aldrich). Cells with integrated genes were selected using 4 μg/ml puromycin (Sigma-Aldrich). Established cells were further grown in Dulbecco’s modified Eagle medium (DMEM) supplemented with 10 % fetal bovine serum (FBS), 100 μg/ml streptomycin, 100 U/ml penicillin, and 1 μg/ml puromycin at 37 °C, under 5 % CO_2_.

Cells with knockdown of autophagy-related gene (ATG)-5 or overexpression of green fluorescence protein (GFP)-microtubule-associated protein-1 light chain 3 (LC3):

Lentiviral vector (pLKO.1) with an insert for short hairpin RNA (shRNA) targeting mouse ATG-5 was provided by the National RNAi Core Facility Platform in Academia Sinica, Taiwan. The accession number of the mouse ATG-5 gene is NM_053069. The control lentivirus and the virus to produce mouse ATG-5 targeting shRNA were made by the RNAi core lab at the Clinical Research Center, National Cheng Kung University Hospital, Tainan, Taiwan. Lentivirus was used to infect mouse NIH/3T3 cells using polybrene (Sigma-Aldrich). Cells with integrated genes were selected using 4 μg/ml puromycin.

To establish cell lines with stable expression of GFP-LC3, control NIH/3T3 cells and NIH/3T3 cells overexpressing IRS-1 were transfected using GFP-LC3 plasmids gifted by Dr. Noboru Mizushima (Tokyo Medical and Dental University, Tokyo, Japan). Following transfection with Lipofectamine 2000 (Invitrogen) for 48 h, positive stable clones were selected by culturing cells with G418 (400 μg/ml) for 2 weeks while being maintained in DMEM supplemented with 10 % FBS, 100 μg/ml streptomycin, 100 U/ml penicillin, and 200 μg/ml G418 at 37 °C, under 5 % CO_2_.

### Detection of intracellular reactive oxygen species (ROS) induced by glucose oxidase (GO)

To investigate the influence of chronic exposure to oxidative stress on autophagy, we used a GO/glucose system as a source of intracellular ROS. Adding GO to the culture medium provides a continuous supply of ROS, and the system is thus a suitable model for studying chronic exposure of cells to ROS [38]. The amount of intracellular ROS in the cytosolic fraction was measured using an OxiSelect™ Intracellular ROS Assay Kit (Cell Biolabs).

### Cell viability and proliferation assay

A trypan blue dye (Invitrogen) exclusion assay was used to examine cell viability. Cells were collected by trypsinization, washed once with phosphate buffered saline (PBS), and suspended in 0.2 % trypan blue solution. Nonviable cells stained with a blue color due to loss of membrane integrity; viable cells excluded the dye and remained unstained (white). The percentage of dead cells was calculated.

Cell proliferation was measured quantitatively by adding 10 % (v/v) alamarBlue (Invitrogen) to the culture medium, according to the manufacturer’s instructions. The reduced form of alamarBlue, an indicator of cell proliferation, was measured using a fluorescence plate reader (SpectralMax M5, Molecular Devices) with excitation and emission wavelengths of 570 nm and 600 nm, respectively.

### Flow cytometry

All cells, including floating and adherent cells, were harvested, washed with PBS, suspended in 1 ml of PBS, and then fixed by adding 3 ml of 100 % ethanol that was cooled to −20 °C in advance. Then, the cells were stored overnight at 4 °C. The cells were washed with PBS and stained with propidium iodide (PI)/Triton X-100 solution (0.1 % Triton X-100, 0.2 mg/ml RNase, 20 μg/μl PI in PBS) for 3 h on ice and in darkness. DNA content was determined by flow cytometry using a FACSCalibur cytometer (BD Biosciences). The percentage of sub-G1 DNA was analyzed by gating on cell cycle dot blots using Windows Multiple Document Interface software (WinMDI) version 2.9.

### Western blot analysis

Cell lysates were prepared using ice-cold lysis buffer (20 mM Tris–HCl at pH 7.5, 1 mM EDTA, 1 mM EGTA, 150 mM NaCl, 1 % Triton X-100, 10 mM NaF, 1 mM Na_3_VO_4_, 10 mM 2-mercaptoethanol, and a protease inhibitors cocktail). The cell lysates were centrifuged at 15,000 rpm for 20 min at 4 °C, and the supernatants were collected for Western blot analysis. The signals of target proteins were detected using a chemiflurorescent-immunoblotting detection reagent (GE HealthCare) and a luminescent image analyzer LAS-1000 (FUJI FILM). Densitometry analysis of Western blots was conducted using Multi Gauge 2.11 software (FUJI FILM), and the expression level of each protein, relative to that of actin, was determined. The following antibodies including anti-p70 ribosomal protein S6 kinase, anti-S6 ribosomal protein, anti-Akt, anti-p44/42 MAPK, anti-glycogen synthase kinase 3 beta, anti-phospho-p70 ribosomal protein S6 kinase (Thr 389), anti-phospho-S6-ribosomal protein (Ser 240/244), anti-phospho-p44/p42 MAPK (Thr 202/Tyr 204), anti-phospho-glycogen synthase kinase 3 beta (Ser 9), anti-phospho-Akt (Thr 308), anti-phospho-Akt (Ser 473), anti-LC3B, anti-ATG5, anti-cleaved caspase 3, and anti-IRS1 were purchased from Cell Signaling Technology. Anti-actin antibody was purchased from Santa Cruz Biotechnology. The Alexa Fluor® 488 goat anti-rabbit IgG was purchased from Invitrogen. Anti-rabbit and anti-mouse secondary antibodies were purchased from Jackson ImmunoResearch Laboratories.

### Fluorescence microscopy

Fluorescence analysis of GFP-LC3:

Cells were seeded in six-well plates over which sterile cover slips had been previously placed. After treatment, the cells were washed twice with PBS and fixed in a solution of 4 % paraformaldehyde and 0.19 % picric acid in PBS for 30 min at room temperature, followed by washing three times with PBS. Finally, slides were mounted with cover slips and examined under a fluorescence microscope (Olympus BX51).

Immunofluorescence analysis of endogenous LC3:

Cells were seeded in six-well plates, over which sterile cover slips had been previously placed. After treatment, the cells were washed twice with TBS and fixed in a solution of 4 % paraformaldehyde and 0.19 % picric acid in PBS for 30 min at room temperature. After washing three times with TBS, the cells were permeabilized in digitonin solution (50 μg/ml digitonin in PBS, pH 7.2) for 5 min at 37 °C. The solution was discarded, and excess digitonin was quenched by incubation in a solution of 50 mM NH_4_Cl in PBS for 5 min at 37 °C. The cells were rinsed twice with TBS and incubated in blocking solution [2 % (w/v) bovine serum albumin and 5 % (v/v) normal goat serum in TBS] for 30 min at 37 °C. After rinsing three times in TBS, the cells were incubated in anti-LC3 antibody solution (5 μg/ml anti-LC3 antibody in blocking solution) for 60 min at 37 °C. The cells were then washed twice with TBS for 5 min each cycle, and incubated in 0.05 % (v/v) goat anti-rabbit IgG conjugated with Alexa488 (Invitrogen), in blocking solution for 60 min at 37 °C, followed by washing five times with TBS for 5 min each wash cycle. Finally, slides were mounted with cover slips and examined under a fluorescence microscope.

### Electron microscopy

The cells to be examined were prefixed in 2 % glutaraldehyde in PBS at 4 °C, treated with 1 % OsO_4_ for 3 h at 4 °C, dehydrated in a series of graded ethanol baths and flat embedded in Epon® epoxy resin. Ultra-thin sections were doubly stained with uranyl acetate and observed under an electron microscope (Hitachi H-7650, 60 kV).

### Statistical analysis

Continuous data are presented as mean averages with standard deviations. Comparison of continuous data was performed by the Student’s T-test or the Mann–Whitney U test using SPSS for WINDOWS, version 12.0. (Chicago, IL, United States). A *p*-value of less than 0.05 (two-tailed) was considered significant.

## Results

### Establishment of NIH/3T3 cells overexpressing functional IRS-1

**Figure 1:**
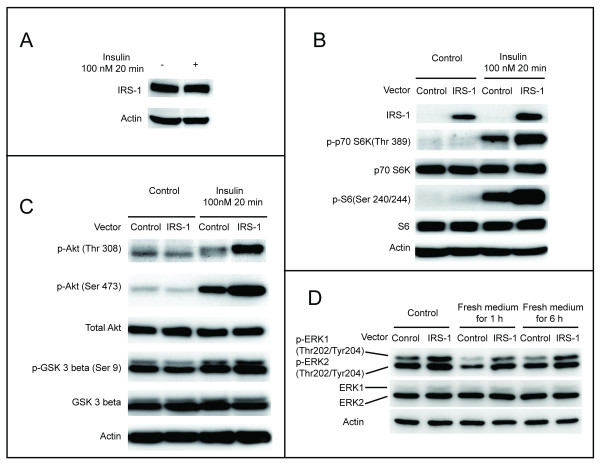
**Establishment of NIH/3T3 cells with stable overexpression of functional IRS-1.** (**A**) Wild-type NIH/3T3 cells were serum starved overnight and then treated with serum free DMEM or with 100 nM insulin in serum free DMEM for 20 min. Endogenous IRS-1 was examined by Western blotting. (**B**) Cells selected after infection by the control retrovirus and those encoding for IRS-1were serum starved overnight and then treated with serum free DMEM or with 100 nM insulin in serum free DMEM for 20 min. The amounts of endogenous and overexpressed IRS-1, and p70 S6K signaling were determined by Western blotting. We used serum free DMEM to avoid the confounding effects on p70 S6K signaling by other growth factors that may be present in serum. (**C**) Cells were serum starved overnight and then treated with serum free DMEM or with 100 nM insulin in serum free DMEM for 20 min. Akt signaling was monitored by Western blotting. Using serum free DMEM avoided the confounding effects of other growth factors that may be present in serum. (**D**) Cells were cultured for 18 h before beginning an experiment. The medium was not changed for the control cell group, but was replaced with fresh complete culture medium for the experimental cell group. The ERK1/2 signaling pathway was analyzed using Western blot analysis.

We chose NIH/3T3 cells as an experimental model to investigate the role of IRS-1 in oxidative stress-mediated autophagy and cell death. Western blotting confirmed the presence of IRS-1 in wild-type NIH/3T3 cells (Figure 1A). To mimic the increased expression levels of IRS-1 seen in tumor cells, we established NIH/3T3 cells with stable overexpression of IRS-1. The levels of total IRS-1 in both the control NIH/3T3 cells and NIH/3T3 cells overexpressing IRS-1 were checked by Western blot analysis. The amount of total IRS-1 was greater in cells infected with retrovirus encoding for the IRS-1 gene than it was in the control cells (Figure 1B), indicating that exogenous IRS-1 was expressed in abundant quantities. Next, we checked if the expressed IRS-1 was functional by determining whether the well-established downstream IRS-1 effectors, including p70 ribosomal protein S6 kinase (p70 S6K), Akt, and ERK were affected by the overexpression of IRS-1. The extent of phosphorylation of p70 S6K at Thr 389, and S6 proteins (the downstream effectors of p70 S6K) at Ser 240/244 was greater in cells overexpressing IRS-1 than in the control cells treated with or without insulin (Figure 1B). Following insulin treatment, the extent of phosphorylation of Akt at Thr 308 and Ser 473, and the extent of glycogen synthesis kinase 3 beta (the downstream effector of Akt) at Ser 9, was greater in the IRS-1 overexpressing cells than it was in the control cells. In the absence of insulin treatment, there were no obvious differences in the extent of phosphorylation of target proteins between the two groups of cells (Figure 1C). The extent of phosphorylation of ERK1 and ERK2 at Thr 202 and Tyr 204 was also greater in cells overexpressing IRS-1 than it was in the control cells under a steady state growth phase (Figure 1D). Thus, we successfully established NIH/3T3 cells with stable overexpression of functional IRS-1 proteins.

### Effect of IRS-1 overexpression on basal autophagy

**Figure 2:**
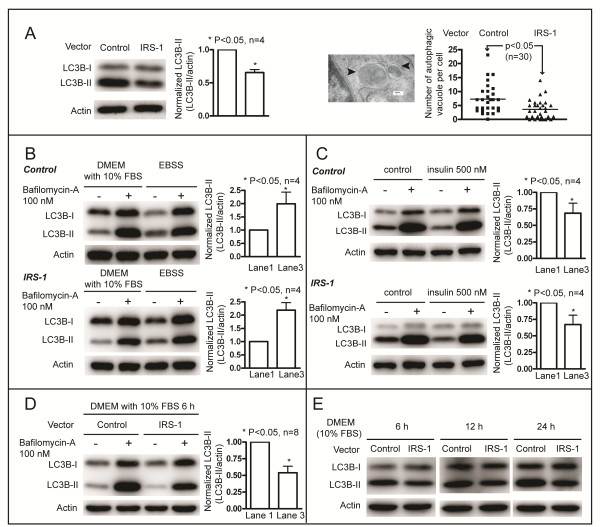
**Effect of IRS-1 overexpression on autophagy.** (**A**) Cells were seeded and cultured for one day. Then, the amounts of LC3B-II between the IRS-1 overexpressing cells and the control cells were compared by Western blotting (left); the level of LC3B-II was normalized to that of actin for comparison (right). Autophagic vacuoles (black arrowheads) were observed under an electron microscope (Scale bar = 100 nm), and were counted in randomly selected groups of 30 cells. (**B**) Cells seeded and cultured for one day were further incubated in fresh DMEM containing 10 % FBS, or in EBSS, an amino acid deficient medium, for 6 h in the presence or absence of 100 nM bafilomycin-A. The influence of amino acid deprivation on autophagy was determined from LC3B-II levels by Western blotting (Left). The levels of LC3B-II in the absence of bafilomycin-A were normalized to that of actin for comparison (right). (**C**) Cells seeded and cultured for one day were further incubated in fresh serum free DMEM with or without 500 nM insulin for 6 h in the presence or absence of 100 nM bafilomycin-A. Using serum free DMEM avoided the confounding effects on autophagy of other growth factors that may be present in serum. The influence of insulin stimulation on autophagy was determined from LC3B-II levels using Western blotting (Left); the levels of LC3B-II in the absence of bafilomycin-A were normalized to that of actin for comparison (right). (**D**) Cells seeded and cultured for one day were further incubated in fresh DMEM containing 10 % FBS for 6 h in the presence or absence of 100 nM bafilomycin-A. LC3B-II levels were determined by Western blotting (Left), and the levels of LC3B-II in the absence of bafilomycin-A were normalized to that of actin for comparison (right). (**E**) Cells seeded and cultured for one day were further incubated in fresh DMEM containing 10 % FBS for the indicated time, and LC3B-II levels were examined by Western blotting.

IRS-1 increases the activity of class I PI3K/Akt signaling and mTOR [31,36], which is located downstream of the class I PI3K/Akt signaling pathway, and is the core negative regulator of autophagy. Thus, it is possible that autophagy is inhibited in NIH/3T3 cells that overexpress IRS-1. To confirm this hypothesis, we investigated basal autophagy by following the conversion of LC3B, from LC3B-I, which is found in the cytosol as a free form, to LC3B-II via conjugation with phosphatidylethanolamine. LC3B-II associates with autophagosome membranes, and its generation is a promising autophagosomal marker [39,40]; the amount of LC3-II usually correlates well with the number of autophagosomes [40]. We compared the induction of autophagy between samples using the LC3B-II level, rather than the LC3B-II:LC3B-I ratio, in accord with suggestions in previous report [41]. We checked cellular levels of LC3B-II during the exponential growth phase, and at roughly 70–80 % confluence, and found that LC3B-II levels in the IRS-1 overexpressing cells were decreased compared to the control cells. Further, we counted the number of autophagic vacuoles visible under an electron microscope. The number of autophagic vacuoles was greater in the control cells than in the IRS-1 overexpressing cells (7.3 + 5.1 *vs*. 3.6 + 3.4, p = 0.002) (Figure 2A). These results indicate that overexpression of IRS-1 reduces the number of autophagosomes, and imply that overexpression of IRS-1 reduces autophagy.

LC3B-II accumulation can result from increased upstream autophagosome formation or from impaired downstream autophagosome-lysosome fusion. To distinguish between these two possible explanations for the decrease in LC3B-II levels in NIH/3T3 cells that overexpress IRS-1, we determined the autophagic flux using LC3 turnover assay in the presence of bafilomycin-A. If the amount of LC3B-II further accumulates in the presence of bafilomycin-A, it indicates that autophagic flux is intact, however, if the LC3B-II levels remain unchanged, it is likely that the autophagic flux is impaired [41,42]. Autophagic flux is used to denote the dynamic processes of autophagosome synthesis, delivery of autophagic substrates to the lysosome, and degradation of autophagic substrates within the lysosome, and is a reliable indicator of autophagic activity [42]. First, we studied the nutrient starvation induced autophagy [43] in both the control cells and the IRS-1 overexpressing cells. Both groups of cells were seeded and cultured for one day, then the culture medium was replaced with fresh DMEM containing 10 % FBS or with Earle’s Balanced Salt Solution (EBSS), an amino acid deficient solution, for 6 h. Treatment with EBSS resulted in increased LC3B-II levels in both the control cells and the IRS-1 overexpressing cells (Figure 2B). The levels of LC3B-II were greater in the presence of bafilomycin-A than in the absence of bafilomycin-A for both groups of cells, either treated with DMEM containing 10 % FBS or with EBSS, indicating that the autophagy fluxes were intact in both groups of cells. We next investigated the effect of insulin, which inhibits autophagy [31,36], on autophagy in both the control cells and the IRS-1 overexpressing cells (Figure 2C). Treatment with 500 nM insulin for 6 h decreased the levels of LC3B-II in both groups of cells. The levels of LC3B-II were greater in the presence of bafilomycin-A than in the absence of bafilomycin-A for both groups of cells either without or with insulin treatment. This finding indicates that the autophagic fluxes remain intact in both the control cells and the IRS-1 overexpressing cells.

We further investigated whether overexpression of IRS-1 inhibits autophagy in this series of experiments. During the exponential growth phase, and at roughly 70 %-80 % confluence, both groups of cells were treated with fresh DMEM containing 10 % FBS, in the absence or presence of bafilomycin-A, for 6 h. As shown in Figure 2D, in the absence of bafilomycin-A, LC3B-II levels in the IRS-1 overexpressing cells were lower than those in the control cells, indicating that there were fewer autophagosomes in the IRS-1 overexpressing cells. The levels of LC3B-II were greater in the presence of bafilomycin-A than in the absence of bafilomycin-A for both groups of cells, indicating that autophagic fluxes are intact in both groups of cells. Further, there was a greater increase in LC3B-II levels between the absence and presence of bafilomycin-A for the control cells than there was for the IRS-1 overexpressing cells, indicating that the autophagic flux was greater in the control cells than in the cells that overexpress IRS-1 [42]. To confirm the decrease of LC3B-II in cells overexpressing IRS-1 during the steady state growth phase, we investigated LC3B-II levels at various times after replacement of the culture medium. Throughout the 24 h monitoring period, LC3B-II levels were lower in cells overexpressing IRS-1 than those were in the control cells (Figure 2E). Taken together, overexpression of IRS-1 inhibits basal autophagy during the steady state growth phase.

### GO increases intracellular ROS and induces autophagy

**Figure 3:**
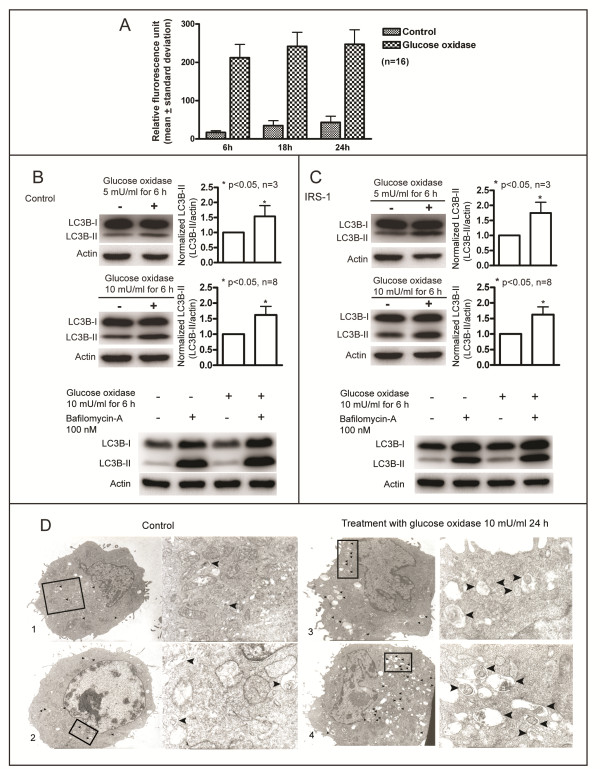
**GO treatment increases intracellular ROS and induces autophagy.** (**A**) Wild-type NIH/3T3 cells were treated with 10 mU/ml GO for various time intervals, and intracellular ROS levels were measured. (**B**) & (**C**) Control cells and cells overexpressing IRS-1 were treated with 5 or 10 mU/ml of GO for 6 h. LC3B-II levels were determined by Western blotting (left), and the levels of LC3B-II were normalized to that of actin for comparison (right) (upper two panels). Bafilomycin-A (100 nM) was added to determine the autophagic flux integrity (bottom panels). (**D**) Wild-type NIH/3T3 cells without (1,2) or with (3,4) treatment of 10 mU/ml GO for 24 h were examined under an electron microscope. Black arrowheads indicate autophagic vacuoles accumulated in cytoplasm.

We first demonstrated that GO actually increases ROS in cells. Wild-type NIH/3T3 cells were either treated with GO (10 mU/ml) or not, and the intracellular ROS was determined. As shown in Figure 3A, an increase in intracellular ROS occurred at 6 h, and lasted for at least 24 h following treatment with GO.

We investigated whether increases in ROS induce autophagy by monitoring changes in LC3B-II levels in response to GO treatment for the control cells (Figure 3B) and the IRS-1 overexpressing cells (Figure 3C). LC3B-II levels in the two groups of cells increased following treatment with GO (5 and 10 mU/ml) for 6 h. The levels of LC3B-II were greater in the presence of bafilomycin-A than in the absence of bafilomycin-A for both the control cells and the IRS-1 overexpressing cells. These results suggest that GO induces autophagy in both groups of cells.

**Figure 4:**
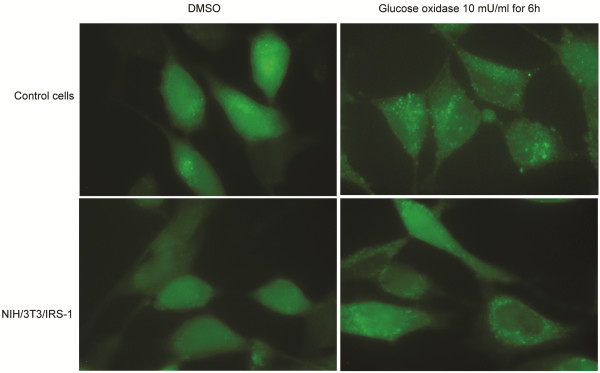
**GO treatment induces autophagy.** Cells expressing GFP-LC3 generated using the controls (upper half) and cells overexpressing IRS-1 (lower half) were treated with 10 mU/ml GO or vehicle (DMSO) for 6 h, and observed under a fluorescence microscope.

Electron microscopy was used to examine GO induced autophagy. During the basal growth state, there were few autophagic vacuoles present in the cytoplasm (Figure 3D, 1 and 2). The numbers of autophagic vacuoles increased after 24 h treatment with GO (10 mU/ml) (Figure 3D and 4). These results indicate that treatment with GO induces autophagy in NIH/3T3 cells.

**Figure 5:**
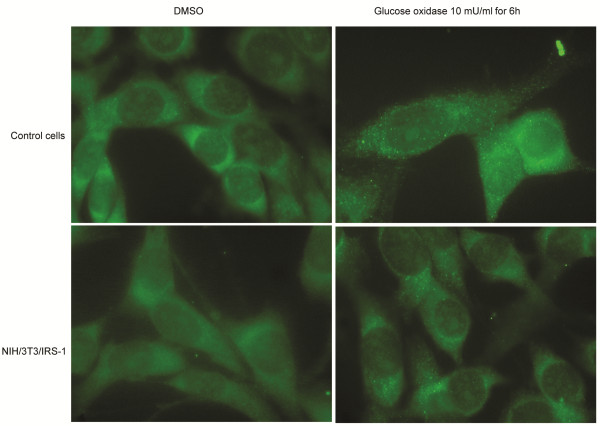
**GO treatment induces autophagy.** Control cells (upper half) and cells overexpressing IRS-1 (lower half) were treated with 10 mU/ml GO or vehicle (DMSO) for 6 h, stained with anti-LC3B antibody and goat anti-rabbit IgG conjugated with Alexa488, and observed under a fluorescence microscope.

We examined the aggregation of GFP-LC3 protein using fluorescence microscopy, to confirm that GO induces autophagy. Upon induction of autophagy, LC3 protein is processed, lipidated, and incorporated into the expanding autophagosome membrane [44]. GFP-LC3 protein is frequently used as an autophagy marker; it translocates from a mainly cytosolic to a punctuate localization upon autophagosome accumulation. There were more green dots in cells treated with GO than there were in cells not receiving GO treatment, for both the control cells and the cells overexpressing IRS-1 (Figure 4). Similar results were observed when the aggregation of endogenous LC3 protein was directly stained with the anti-LC3 antibody and the Alexa488-conjugated secondary antibody (Figure 5). These results further support that GO induces autophagy.

### IRS-1 reduces oxidative stress-mediated autophagy

We hypothesized that oxidative stress induces autophagy via inhibition of IRS-1/Akt/mTOR signaling, and that enhancement of the IRS-1/Akt/mTOR signaling would reduce oxidative stress-mediated autophagy. We examined the phosphorylation of p70 S6K at Thr 389 as a representative of mTOR activity, because p70 S6K is the main downstream effector of mTOR.

**Figure 6:**
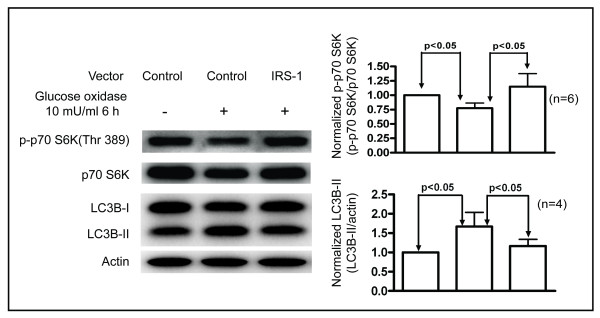
**IRS-1 reduces the oxidative stress-mediated autophagy.** Control cells and cells overexpressing IRS-1 were treated with vehicle (DMSO) or 10 mU/ml GO for 6 h. Changes in the extent of phosphorylation of p70 S6K at Thr 389 and in LC3B-II levels were analyzed by Western blotting (left). The levels of phosphorylated p70 S6K and LC3B-II were normalized to that of actin for comparison (right).

After treatment with GO, LC3B-II levels were increased and the extent of phosphorylation of p70 S6K at Thr 389 was reduced in the control cells (Figure 6). These results confirm that oxidative stress reduces mTOR activity and induces autophagy. In cells overexpressing IRS-1, the influence of GO on LC3B-II levels and phosphorylation of p70 S6K at Thr 389 was lessened. These results suggest that overexpression of IRS-1 attenuates the inhibition of mTOR/p70 S6K activity that is induced by treatment with GO, and restores the ability of mTOR to regulate autophagy.

### Effect of IRS-1 on oxidative stress-mediated cell fate

**Figure 7:**
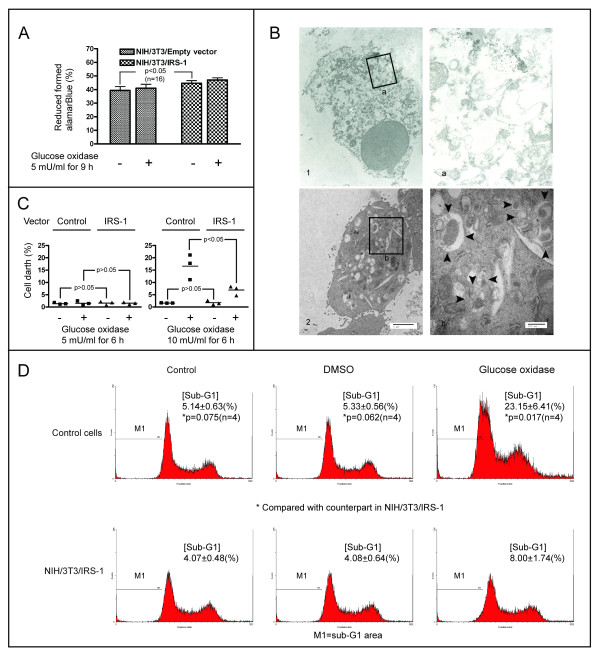
**Effect of IRS-1 on oxidative stress-mediated cell fate.** (**A**) Control cells and cells overexpressing IRS-1 were treated with 5 mU/ml GO for 9 h. Cell proliferation was measured using alamarBlue assay. (**B**) Wild-type NIH/3T3 cells were treated with 10 mU/ml GO for 24 h. Dead cells were observed using an electron microscope. The scale bar in panel B-2 represents 2 μm, and in B-b, represents 500 nm. Black arrowheads indicate autophagic vacuoles. (**C**) Control cells and cells overexpressing IRS-1 were treated with 5 or 10 mU/ml GO for 6 h. Cells were collected by trypsinization and stained with trypan blue. Dead cells were observed and counted under a light microscope. Three dishes of cells of each condition were observed for statistical analysis. Results from a representative of three independent experiments are shown. (**D**) Control cells and cells overexpressing IRS-1 were treated with 10 mU/ml GO for 6 h. Cells were collected by trypsinization and stained with PI. Cell death was analyzed from the proportion of sub-G1 group DNA determined by flow cytometry analysis. Four independent experiments were performed for statistical analysis. M1 indicates the sub-G1 DNA area.

Low levels of ROS promote cell growth, but high levels induce cell death [13]. We have shown above that IRS-1 reduces oxidative stress mediated-autophagy. Although autophagy usually serves as a survival mechanism, excessive autophagy may lead to cell death [45,46]. We studied the effect of IRS-1 on oxidative stress-mediated cell fate by using the control cells and NIH/3T3 cells overexpressing IRS-1 (Figure 7A). The quantity of the reduced form of alamarBlue, an indicator of cell proliferation, was greater in cells overexpressing IRS-1 compared to that in the control cells, indicating that IRS-1 promotes cell proliferation. In addition, the amount of the reduced form of alamarBlue was slightly greater in cells treated with 5 mU/ml GO than that in cells without treatment, for both the control cells and the IRS-1 overexpressing cells, indicating that low levels of oxidative stress (5 mU/ml GO) promoted cell proliferation. However, high levels of oxidative stress (10 mU/ml GO) resulted in cell death, manifested by rounding of the cells, and detachment of the cells from the culture dish.

We used electron microscopy to observe the morphologies of cells that perished due to high ROS levels. Wild-type NIH/3T3 cells were treated with 10 mU/ml GO for 24 h. All cells, whether floating in the medium, or attached to the culture dish, were collected and prepared for electron microscopy. As shown in Figure 7B-1, the cells manifested characteristics of necrosis, including swollen cells and mitochondria, disruption of the cellular membrane, and cell lysis [47]. Autophagic vacuoles had accumulated in the dying cells (Figure 7B-2), indicating that oxidative stress-mediated cell death is accompanied by induction of autophagy. We further compared the extent of cell death caused by ROS for the control cells and the IRS-1 overexpressing cells. The control cells, and cells overexpressing IRS-1, were treated with 5 and 10 mU/ml GO for 6 h. Cells were collected by trypsinization and stained with trypan blue. The proportion of cell death was similar for both groups of cells during the basal growth state. GO treatment at 5 mU/ml did not result in cell death; however, cell death ensued from GO treatment at 10 mU/ml, with a lower percentage of mortality in cells overexpressing IRS-1 than that seen for the controls (Figure 7C). We used flow cytometry assay to confirm that IRS-1 provides protection against cell death caused by oxidative stress [48]. The control cells and the IRS-1 overexpressing cells were treated with 10 mU/ml GO for 6 h. The cells were collected using trypsinization and stained with PI for flow cytometry analysis [48]. The high levels of oxidative stress induced less cell death in cells overexpressing IRS-1 than it did in the control cells (Figure 7D). Taken together, overexpression of IRS-1 promotes cell growth and reduces oxidative stress mediated-cell death.

### Oxidative stress induces autophagy-dependent cell death

**Figure 8:**
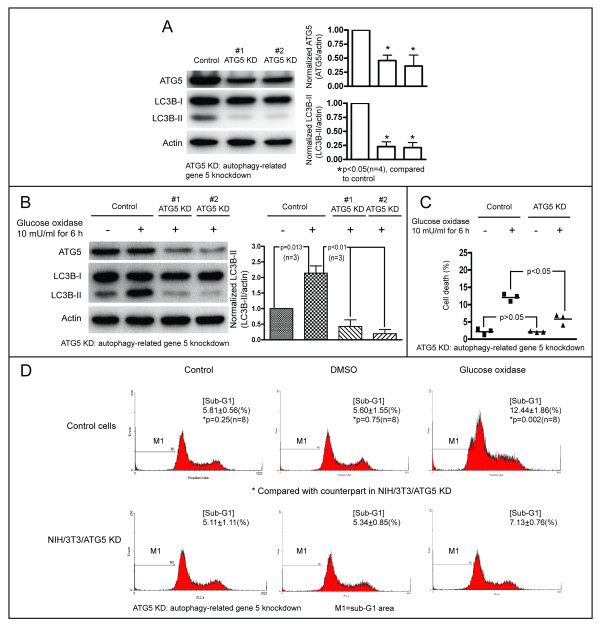
**Oxidative stress induces autophagy-dependent cell death.** (**A**) Two stable ATG-5 knockdown cell lines were examined for the presence of ATG-5 by Western blotting (left). The levels of ATG-5 and LC3B-II were normalized to that of actin for comparison over four independent experiments (right). (**B**) Control cells and the ATG-5 knockdown cells were treated with DMSO or GO for 6 h. ATG-5, LC3B-I, and LC3B-II levels were examined by Western blotting (left). The levels of LC3B-II were normalized to that of actin for comparison, using three independent experiments (right). (**C**) Control cells and the ATG-5 knockdown cells were treated with 10 mU/ml GO for 6 h. Cell death was examined using trypan blue dye exclusion assay. Three dishes of cells of each condition were observed for statistical analysis. The results for a representative sample of three independent experiments are shown. (**D**) Control cells and the ATG-5 knockdown cells were treated with 10 mU/ml GO for 6 h. Cells were collected using trypsinization and stained with PI. Cell death was analyzed from the proportional area of sub-G1 group DNA, as determined by flow cytometry. Eight independent experiments were performed for statistical analysis. M1 indicates the sub-G1 area.

Our electron microscopy observations of cell death confirmed that oxidative stress induces cell necrosis. However, the manifestations of cell morphologies characteristic of cell necrosis suggest necrotic cell death, apoptotic cell death with secondary necrosis, or autophagic cell death. Oxidative stress induces autophagy, and excess autophagy causes cell death [45,46]; cell death caused by GO treatment is accompanied by induction of autophagy (Figure 7B-2). Thus, we wondered whether oxidative stress induces autophagy-dependent or autophagic cell death in the NIH/3T3 cells used in this study. To answer this question, we investigated whether inhibition of autophagy by knockdown of ATG-5 affects GO-induced cytotoxicity in NIH/3T3 cells for determining autophagic cell death [49]. Wild-type NIH/3T3 cells were infected with lentivirus containing an insert encoding shRNA for ATG-5, to establish stable NIH/3T3 cell lines with knockdown of ATG-5. As shown in Figure 8A, ATG-5 levels were reduced in the two stable cell lines, and the levels of LC3B-II, an indicator of autophagy induction, were reduced by roughly 75 % in both the two stable cell lines. These results confirm that knockdown of ATG-5 was successful and autophagy was reduced in these knockdown cells. The control cells and the ATG-5 knockdown cells were trea-ted with 10 mU/ml GO for 6 h. As anticipated, the treatment resulted in increased LC3B-II levels in the control cells, and this effect was reversed in the ATG-5 knockdown cells (Figure 8B). Cell death following treatment with 10 mU/ml GO for 6 h was analyzed by trypan blue dye exclusion assay and flow cytometry. The proportion of cell death was similar for both the control cells and the ATG-5 knockdown cells during the basal growth state. However, there was a lower percentage of cell death seen for the ATG-5 knockdown cells than that for the control cells, following treatment with 10 mU/ml GO for 6 h (Figure 8C). Flow cytometry analysis showed no differences in the sub-G1 peak between the control cells and the ATG-5 knockdown cells in the absence of GO treatment; however, following treatment with 10 mU/ml GO, the sub-G1 peak area was less in the ATG-5 knockdown cells than it was in the controls (Figure 8D). Taken together, these results indicate that autophagy induction by oxidative stress does not protect cells from death, and that oxidative stress induces autophagy-dependent or autophagic cell death.

## Discussion

The current study shows that overexpression of IRS-1 promotes cells growth, inhibits basal autophagy, reduces oxidative stress-induced autophagy, and diminishes oxidative stress-mediated autophagy-dependent cell death. We have provided evidence that ROS induces autophagy via inhibition of IRS-1/PI3K/mTOR signaling.

We found that low levels of ROS promote cell proliferation (Figure 7A), while high levels induce cell death, in agreement with previous reports [13]. We found that the flow cytometry sub-G1 peak area increased in the DNA histogram (Figure 7D, Figure 8D), indicating that ROS induces apoptosis, and that GO generated ROS induced autophagy (Figure 3B-D, Figure 4, Figure 5). Oxidative stress-induced autophagy did not protect cells from death; inhibition of autophagy by knockdown of ATG-5 reduced cell death caused by oxidative stress (Figure 8C-D). These data suggest that oxidative stress induces autophagy-dependent or autophagic cell death. Autophagy has been proposed to kill the cells directly, and to participate in a lethal signaling event activating an apoptotic or necrotic death pathway [50]. Our data is consentient with other reports [51-53] supporting the notion that “autophagic cell death” does occur, although it is often thought to be a misnomer [47]. Indeed, there are numerous reports suggesting that autophagy is a survival mechanism that protects cells in response to environmental stresses. In human and mouse cells, deletion of autophagy-related genes generally fails to confer protection against the induction of cell death by stressors, and rather accelerates cell death [54,55]. Additionally, the observation that chemicals with the ability to inhibit autophagy significantly accelerate cellular necrosis further supports the idea that autophagy acts primarily as a cytoprotective, rather than cytotoxic process [56]. In summary, oxidative stress can cause necrotic, apoptotic, and autophagic cell death.

Our observation of reduced phosphorylation of p70 S6K, a major downstream effector of mTOR, in response to GO treatment indicates that oxidative stress reduces mTOR activity (Figure 6). Additionally, overexpression of IRS-1 attenuates the inhibitory effect of oxidative stress on mTOR/p70 S6K signaling (Figure 6). These results suggest that overexpression of IRS-1 competes with the inhibitory signal mediated by oxidative stress on mTOR. Importantly, the oxidative stress mediated induction of autophagy was attenuated by overexpression of IRS-1 (Figure 6). Taken together, these findings suggest that inhibition of IRS-1/PI3K/Akt/mTOR signaling is another mechanism for oxidative stress-induced autophagy.

We demonstrated that overexpression of IRS-1 inhibits autophagy in the present study. The previous finding indicating that knockout of IRS-1 results in increased numbers of autophagosomes in mice cardiomyocytes [57] further supports our data, and suggests that IRS-1 is involved in the regulation of autophagy. We found that overexpression of IRS-1 increases both ERK and mTOR/p70 S6K activity (Figure 1B, Figure 1D). Activation of ERK signaling induces autophagy [58], activation of mTOR signaling inhibits autophagy [29], and activation of p70 S6K signaling induces autophagy [59,60]. Basal autophagy was decreased in cells overexpressing IRS-1 (Figure 2) even though ERK and p70 S6K signaling were activated. This might be due to the interaction of complex intracellular signaling networks in response to different stimuli, and be explained by the presence of different downstream mTOR signaling pathways. The mTOR/p70 S6K signaling is involved in cell growth, thus, cells overexpressing IRS-1 grow more rapidly than the control cells do (Figure 7A). However, the mTOR/unc-51-like kinase (ULK) signaling negatively regulates autophagy [31]. In summary, mTOR is activated by overexpression of IRS-1 in cells, in which autophagy is inhibited.

Despite its lack of intrinsic kinase properties, IRS-1 is thought to be involved in tumorigenesis, it interacts with β-catenin, an important regulator of stem/progenitor cell fate, and levels of β-catenin target genes, such as c-myc and cyclin D1, are increased in mammary tumors that overexpress IRS-1 [61]. IRS-1 directly binds, interacts, and cooperates with numerous oncogene proteins, including JCV T antigen [62], and simian virus 40 T antigen [63]. Additionally, IRS-1 has an anti-apoptotic function that protects cells from apoptotic cell death [64,65]. In this study, we found that activation of IRS-1 signaling promotes cell proliferation (Figure 7A), probably via concomitant activation of mTOR/p70 S6K and ERK signaling (Figure 1B & D). Both of these pathways are involved in cell growth and proliferation [4]. Further, IRS-1 protects cells from oxidative stress-mediated cell death (Figure 7C, D). These may be the reasons why the expression levels of IRS-1 increase in some types of cancers. Thus, our findings afford a credible explanation for IRS-1 involvement in the tumor initiation and progression.

**Figure 9:**
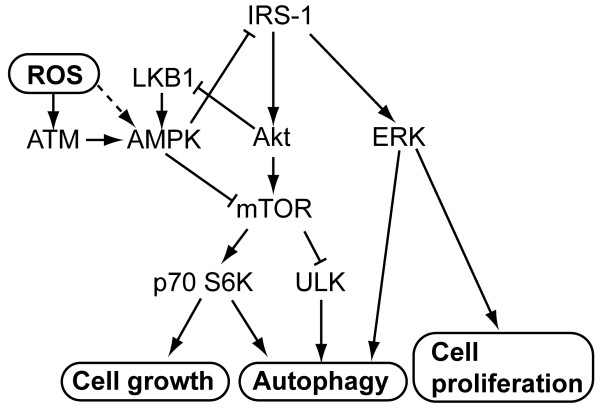
**The interaction between IRS-1 and oxidative stress in the regulation of autophagy and cell growth.** The reported signaling pathways and the results presented in this study are summarized.

The proposed relationship between IRS-1, oxidative stress, and regulation of autophagy and cell growth is shown in Figure 9. In addition to activating p70 S6K to promote cell growth, mTOR negatively regulates autophagy via inhibition of the ULK complex. IRS-1 promotes cell growth and inhibits autophagy by enhancing mTOR activity; it also promotes cell proliferation via activation of ERK signaling. ROS activates AMPK by activating ATM protein, or via other pathways; AMPK then promotes autophagy through direct inhibition of mTOR, or by indirect inhibition of IRS-1/Akt/mTOR signaling. By contrast, IRS-1 can reduce AMPK activity by inhibition of LKB1. Both the ERK [58] and p70 S6K [59,60] signaling can induce autophagy.

## Conclusion

Our results imply that IRS-1 plays a poorly defined but important role in the pathogenesis of human diseases that exhibit abnormal proliferation of cells, such as cancers, benign prostate hyperplasia, and atherosclerotic coronary artery disease. This is because IRS-1 can promote cell proliferation and help cells to resist the oxidative stresses generated during cell proliferation. Further investigation into the role of the IRS-1 protein in specific human diseases that feature increased expression levels of IRS-1 would be worthwhile. Genetic or pharmacologic intervention to inhibit IRS-1 signaling might be an effective strategy to treat diseases characterized by uncontrolled proliferation of cells.

## Abbreviations

IRS, Insulin receptor substrate; ROS, Reactive oxygen species; GO, Glucose oxidase; LC3, Microtubule-associated protein 1 light chain 3; mTOR, Mammalian target of rapamycin; p70 S6K, p70 ribosomal protein S6 kinase; PI3K, Phosphatidylinositol 3-kinase; Akt/PKB, Protein kinase B; MAPK, Mitogen activated protein kinase; ERK, Extracellular signal-regulated kinase; AMPK, AMP-activated protein kinase; ATM, Ataxia-telangiectasia mutated; LKB, Liver kinase B; DMEM, Dulbecco’s modified Eagle medium; ATG-5, Autophagy-related gene 5; GFP, Green fluorescence protein; PBS, Phosphate buffered saline; TBS, Tris-buffered saline; PI, Propidium iodide; EBSS, Earle’s Balanced Salt Solution; ULK, Unc-51-like kinase; FBS, Fetal bovine serum; shRNA, Short hairpin RNA; DMSO, Dimethyl sulfoxide; GSK, Glycogen synthase kinase.

## Competing interests

The authors declare no potential conflict of interests.

## Authors’ contributions

Dr. Shih-Hung Chan conceived this study and conducted experiments. Dr. Hidenori Matsuzaki afforded great help in the experiments. Prof. Jyh-Hong Chen participated in the coordination of the study. Prof. Ushio Kikkawa helped to revise the manuscript. Prof. Wen-Chang Chang conceived the study and critically revised the manuscript. All authors read and approved the final manuscript.
